# A hypolipoprotein sepsis phenotype indicates reduced lipoprotein antioxidant capacity, increased endothelial dysfunction and organ failure, and worse clinical outcomes

**DOI:** 10.1186/s13054-021-03757-5

**Published:** 2021-09-17

**Authors:** Faheem W. Guirgis, Lauren Page Black, Morgan Henson, Guillaume Labilloy, Carmen Smotherman, Charlotte Hopson, Ian Tfirn, Elizabeth L. DeVos, Christiaan Leeuwenburgh, Lyle Moldawer, Susmita Datta, Todd M. Brusko, Alexis Hester, Andrew Bertrand, Victor Grijalva, Alexander Arango-Esterhay, Frederick A. Moore, Srinivasa T. Reddy

**Affiliations:** 1 Department of Emergency Medicine, University of Florida College of Medicine – Jacksonville, 655 West 8th Street, Jacksonville, FL 32209 USA; 2 Center for Data Solutions, University of Florida College of Medicine – Jacksonville, Jacksonville, FL USA; 3 Department of Aging and Geriatric Research, University of Florida College of Medicine, Gainesville, FL USA; 4 Department of Surgery, University of Florida College of Medicine, Gainesville, FL USA; 5 Department of Biostatistics, University of Florida, Gainesville, FL USA; 6 Department of Pathology, Immunology, and Laboratory Medicine, University of Florida Diabetes Institute, College of Medicine, Gainesville, FL USA; 7 Department of Pediatrics, University of Florida Diabetes Institute, College of Medicine, Gainesville, FL USA; 8 Division of Cardiology, Department of Medicine, David Geffen School of Medicine at UCLA, Los Angeles, CA USA

**Keywords:** Sepsis, Lipids, Lipoprotein, Shock, Inflammation, Quality of life

## Abstract

**Objective:**

Approximately one-third of sepsis patients experience poor outcomes including chronic critical illness (CCI, intensive care unit (ICU) stay > 14 days) or early death (in-hospital death within 14 days). We sought to characterize lipoprotein predictive ability for poor outcomes and contribution to sepsis heterogeneity.

**Design:**

Prospective cohort study with independent replication cohort.

**Setting:**

Emergency department and surgical ICU at two hospitals.

**Patients:**

Sepsis patients presenting within 24 h.

**Methods:**

Measures included cholesterol levels (total cholesterol, high density lipoprotein cholesterol [HDL-C], low density lipoprotein cholesterol [LDL-C]), triglycerides, paraoxonase-1 (PON-1), and apolipoprotein A-I (Apo A-I) in the first 24 h. Inflammatory and endothelial markers, and sequential organ failure assessment (SOFA) scores were also measured. LASSO selection assessed predictive ability for outcomes. Unsupervised clustering was used to investigate the contribution of lipid variation to sepsis heterogeneity.

**Measurements and main results:**

172 patients were enrolled. Most (~ 67%, 114/172) rapidly recovered, while ~ 23% (41/172) developed CCI, and ~ 10% (17/172) had early death. ApoA-I, LDL-C, mechanical ventilation, vasopressor use, and Charlson Comorbidity Score were significant predictors of CCI/early death in LASSO models. Unsupervised clustering yielded two discernible phenotypes. The Hypolipoprotein phenotype was characterized by lower lipoprotein levels, increased endothelial dysfunction (ICAM-1), higher SOFA scores, and worse clinical outcomes (45% rapid recovery, 40% CCI, 16% early death; 28-day mortality, 21%). The Normolipoprotein cluster patients had higher cholesterol levels, less endothelial dysfunction, lower SOFA scores and better outcomes (79% rapid recovery, 15% CCI, 6% early death; 28-day mortality, 15%). Phenotypes were validated in an independent replication cohort (N = 86) with greater sepsis severity, which similarly demonstrated lower HDL-C, ApoA-I, and higher ICAM-1 in the Hypolipoprotein cluster and worse outcomes (46% rapid recovery, 23% CCI, 31% early death; 28-day mortality, 42%). Normolipoprotein patients in the replication cohort had better outcomes (55% rapid recovery, 32% CCI, 13% early death; 28-day mortality, 28%) Top features for cluster discrimination were HDL-C, ApoA-I, total SOFA score, total cholesterol level, and ICAM-1.

**Conclusions:**

Lipoproteins predicted poor sepsis outcomes. A Hypolipoprotein sepsis phenotype was identified and characterized by lower lipoprotein levels, increased endothelial dysfunction (ICAM-1) and organ failure, and worse clinical outcomes.

**Supplementary Information:**

The online version contains supplementary material available at 10.1186/s13054-021-03757-5.

## Introduction

Sepsis is a prevalent, morbid and costly condition. In 2017, there were an estimated 48.9 million cases worldwide, with 11 million sepsis-related deaths accounting for 19.7% of all global deaths [1]. In the United States, there are approximately 1.7 million annual sepsis cases with an overall mortality of nearly 20% [2–4]. {Formatting Citation} Sepsis patients who survive hospital admission frequently experience poor quality-of-life and increased long-term mortality [5, 6]. Nearly one-third of sepsis patients develop a state of chronic critical illness (CCI), defined as intensive care unit (ICU) stay ≥ 14 days with continued organ dysfunction. They are characterized by lean muscle wasting, cachexia, and a 1-year mortality approaching 50% [6–8]. CCI patients require high levels of post-discharge care, and most of them are discharged to skilled nursing facilities or long-term acute care hospitals. Studying chronic drivers of inflammation and dysregulated immunity is key to understanding these poor outcomes.

Lipids and lipoproteins play an important role in sepsis pathogenesis. Studies have shown that high density lipoprotein cholesterol (HDL-C) and low density lipoprotein cholesterol (LDL-C) are protective in sepsis [9, 10]. First, HDL has antioxidant and anti-inflammatory functions mediated by paraoxonase 1 (PON-1, an HDL-associated esterase that protects lipids from oxidation) and apolipoprotein A-I (Apo A-I) [11, 12]. ApoA-I has been shown to suppress both neutrophil and monocyte activation [13–15]. HDL has also been shown to downregulate inflammatory pathways in sepsis via the transcriptional regulator ATF3 [16]. Second, both HDL-C (via ApoA-I) and LDL-C bind and clear bacterial toxins during sepsis [11, 17–23]. Third, HDL has direct endothelial protective effects during sepsis [24, 25].

Changes in lipid and lipoprotein levels, structure, and function may affect their ability to protect against sepsis. Barlage and colleagues demonstrated several lipoprotein differences between survivors and non-survivors in sepsis including that non-survivors had lower levels of total cholesterol, LDL-C, HDL-C, Apo-A-I, and Apo-B at all time points (day 1, 4, and 11) [26]. ApoA-I levels were an independent predictor of mortality in their cohort after adjusting for other factors, and HDL-C and ApoA-I levels were depressed and stayed the same or decreased in non-survivors, compared with survivors in whom levels gradually increased over the course of their hospitalization. Barlage and colleagues also found that HDL mediators of monocyte activity such as Apo-CI (which stimulates the response to LPS by macrophages), were reduced in non-survivors. More recently, others have shown a close clinical correlation between early HDL-C levels in sepsis and severity of organ failure or survival [27, 28], that HDL-C levels correlate inversely with pro-inflammatory cytokines including TNF-α, IL-6, IL-10 [28], and that cholesterol levels three days after sepsis admission do not return to pre-sepsis baseline levels [29]. Reasons for reduced HDL-C levels in sepsis may include decreased hepatic synthesis, consumption as part of the systemic response to infection, or increased clearance via scavenger receptor B1 [30].

Genetic studies designed to evaluate for causality identified an important link between genetically determined HDL-C levels and decreased risk of hospitalizations for infectious disease, lower odds of outpatient antibiotic usage, and reduced risk of mortality from sepsis, but not for LDL-C, or triglycerides [31, 32]. A rare missense variant in the cholesteryl ester transfer protein (CETP) gene was also recently identified and linked with significant reductions in HDL-C. Carriers of this allele had decreased survival, more severe organ failure and reduced 28-day survival.

We have shown that HDL becomes pro-inflammatory and dysfunctional (Dys-HDL) in sepsis, and that Dys-HDL correlates with and predicts organ failure in sepsis [33–35]. Changes in HDL composition may partly explain the presence of Dys-HDL in sepsis. Some of these changes are due to increased levels of acute phase proteins such as serum amyloid A (SAA) which can displace ApoA-I and contribute to HDL’s pro-inflammatory state and may also contribute to reduced HDL-C levels [36, 37]. Also, HDL particle size is increased in septic patients compared to similarly ill non-septic ICU patients [38]. Larger HDL particles are less functional, and less mature than smaller, more dense HDL.

Given the complexity of lipid and lipoprotein metabolism in sepsis but strong associations with important clinical outcomes, our objectives were two-fold: (1) to confirm the predictive ability of lipids and lipoproteins for the clinically important outcomes of rapid recovery, CCI, and early death previously established by our group, and (2) determine the contribution of lipids and lipoproteins to sepsis heterogeneity. We hypothesized that lipids and lipoproteins would be predictive of poor outcomes, a composite of CCI and early death, from sepsis. To advance our understanding of complex metabolic, endothelial, and inflammatory relationships beyond predicting outcomes, we used use unsupervised clustering methods to phenotype sepsis patients using lipid and lipoprotein data.

## Methods

### Design

This was a two-site, prospective, longitudinal cohort study of sepsis patients enrolled from two locations between November 2016 and June 2018: the emergency department at UF Health Jacksonville and the surgical intensive care unit (ICU) at UF Health Shands (Gainesville). UF Health Shands surgical ICU patients were a subset of a larger study validating the persistent inflammation, immunosuppression, and catabolism syndrome as a mechanism for CCI after sepsis [39]. The studies were approved by the University of Florida Institutional Review Board, and registered with *clinicaltrials.gov* (NCT02934997; NCT02276417). STROBE guidelines for observational studies were followed [40].

### Patient selection and enrollment

UF Health Jacksonville emergency department patients meeting Sepsis-3 criteria were identified prospectively by trained research coordinators or providers within 24 h of sepsis recognition [3]. Patient enrollment occurred seven days per week, between the hours of 7 am and 12 am. Similarly, UF Health Shands Gainesville patients admitted to the surgical ICU and entered into the standard-of-care sepsis protocol were enrolled 24 h/day, seven days a week. For Gainesville patients, Sepsis-2 criteria were used as the study began in 2015 prior to the development of Sepsis-3. Interim analysis of this cohort in 2017 demonstrated that only 7% of study patients that were classified as sepsis by Sepsis-2 would have been reclassified as infection by Sepsis-3 (because of lack of attributable organ dysfunction) [41]. When various equivalent strata of Sepsis-2 and Sepsis-3 cohorts were compared, no significant difference in immune biomarkers, sequential organ failure assessment (SOFA) scores, inpatient clinical outcomes, discharge disposition, mortality or long-term performance status were found. Exclusion criteria were the same for both cohorts: (a) significant traumatic brain injury (evidence of neurologic injury on CT scan and a GCS < 8), (b) refractory shock (likely death within 12 h), (c) alternative/confounding diagnosis causing shock, (d) uncontrollable source of sepsis, (e) patients deemed futile care, (f) severe CHF (NY Heart Association Class IV), (g) Child–Pugh Class B or C liver disease, (h) known HIV with CD4 count < 200 cells/mm3, (i) organ transplant recipient on immunosuppressive agents, (j) known pregnancy, (k) inability to obtain informed consent, and (l) diagnosed disorders of lipid metabolism.

### Enrollment blood sampling and testing

Blood was drawn at the time of enrollment (first 24 h). Testing included cholesterol levels, Dys-HDL measured via HII, PON-1, ApoA-I, inflammatory biomarkers (growth related oncogene (GRO), granulocyte colony stimulating factor (G-CSF), granulocyte macrophage stimulating factor (GM-CSF), interleukin-6 (IL-6), IL-8, IL-10, IL-12p70, macrophage inflammatory protein-1α (MIP-1α), interferon-α (IFN-α), IL-1β, interferon gamma-induced protein (IP-10), monocyte chemotactic protein-1 (MCP-1), IL-10, tumor necrosis factor alpha (TNF-α), and interferon gamma (IFN-γ)), endothelial markers (human intercellular adhesion molecule-1 (ICAM-1) and e-selectin levels and activity). Serum total cholesterol, HDL-C, and triglyceride levels were directly measured from serum samples. LDL-C was calculated using the Friedewald formula [42]. PON-1 activity and HII were measured and reported as in prior studies [43]. Quantikine™ ELISA kits (R&D Systems, Inc, Minneapolis, MN) were used to measure plasma ApoA-I, human ICAM-1 (ICAM-1), and human e-selectin levels and activity. Plasma MPO levels were measured using the ELISA kit from Mercodia (Sweden). Plasma cytokine concentrations were measured using MagPix™ multiplex assay by Luminex (Austin, Texas). Select cytokines were chosen and measured based on previous associations with pro-inflammatory HDL and other lipids [33].

### Data collection

All data were reviewed and entered into a Research Electronic Data Capture (REDCap) database by trained research coordinators [44]. Prospectively collected data included demographics, place of residence, source of infection, and Charlson Comorbidity Score [45]. Clinical variables including triage and enrollment vital signs, SOFA score, timing of antibiotics, volume of intravenous fluids administered in the first six and 24 h, vasopressor use and duration, mechanical ventilation use and duration, urine output in the first six hours, and medications. Admission disposition, hospital length of stay (LOS), and ICU LOS were documented. At 48–96 h, repeat clinical assessments were performed.

### Clinical outcomes and adjudication

The primary outcome was one of three categories: (1) early death (within 2 weeks of sepsis onset), (2) CCI (total ICU stay > 14 days with organ dysfunction or total ICU ≤ 14 days but discharged to long-term acute care, another hospital, or hospice), or (3) rapid recovery (all others) [46]. Group adjudication was performed for the primary outcomes and hospital disposition during sepsis adjudication meetings performed at both sites [39]. Infection type, primary source of infection, and culture positivity was adjudicated by the PI for all cases with at least 10% review by co-investigators and had a kappa of 0.64 (*p* = 0.003). Social security death index was used to determine mortality for patients lost to follow up.

### *Sample size and data analysis (see **Methods** Supplement for details)*

#### Univariate comparisons and regression model

Summary statistics are presented by frequencies and percentages for categorical data, and by medians and quartiles for continuous data. Unadjusted comparisons among groups (rapid recovery, CCI, early death) were performed using the Pearson’s Chi-square test (or Fisher’s exact test if cell frequencies were < 5) for categorical data, and using Wilcoxon rank sum test for continuous data. Correlations between continuous variables were assessed using Spearman’s ρ correlation coefficient. In multivariable analyses, multiple logistic regressions were used to investigate the potential predictive nature of several variables on a binary outcome measure (CCI or early death versus rapid recovery). Least Absolute Shrinkage and Selection Operator (LASSO) variable selection technique was used to identify important predictors of CCI or early death [47]. The following variables were included in the full logistic regression model and entered the LASSO selection: age, gender, race, PON1, ApoA-I, HDL-C, LDL-C, total cholesterol, triglycerides, G-CSF, GM-CSF, IFN-γ, IL-10, IL-12p70, IL-6, IL-8, IP-10, MCP-1, MIP1a, TNF-α, SOFA score, initial lactate, mechanical ventilation use, statin use, vasopressor use, vasopressor duration, volume of intravenous fluids in the first 24 h, APACHE II Score and Charlson Comorbidity Score. Missing data were treated as missing in univariate comparisons and LASSO models. Analyses were performed using SAS® Version 9.4 for Windows (Cary, NC, USA) or R Core Team (2020) (Vienna, Austria).

#### Unsupervised clustering analysis

We used hierarchical agglomerative clustering to identify underlying clusters. We computed the linkage matrix using Spearman's correlation and Ward's Method [48, 49]. From the linkage matrix, we extracted the first two clusters, as these clusters were easily differentiated visually and clinically differed by their primary outcomes. This choice was further justified using the elbow curve and the Calinski–Harabasz score for k-means clustering (Additional file 7: Fig. 7). We labeled the left cluster the “Hypolipoprotein cluster,” and the right cluster the “Normolipoprotein cluster.” We then tested each feature for the difference of means between the two clusters using a two-sided *t*-test. We used this set of *t*-tests as a signature (or set of features) segregating the two outcomes groups with *p* < 0.05 level to determine significance. Significant features at the *p* < 0.05 level were labeled with single asterisk *, and features significant at *p* < 0.0001 were labeled with double asterisks ** in Additional file 10: Table 3. To assess clinical relevance, after extracting clusters, we compared differences in mortality and rates of CCI, early death, and rapid recovery among the derived clusters. We provide visual representation of the unsupervised clustering data using the Seaborn clustermap function to illustrate the differential analysis displaying samples clustered only and features sorted by feature category. Median value imputation was performed for missing values. See *Methods** Supplement* for further details.

### Independent replication cohort

The unsupervised clustering signature derived in our primary cohort, was then tested for validation in a secondary, independent replication cohort of patients. Details on this cohort were published previously [50]. Briefly, the replication cohort study included samples and data from 86 patients from a prior, prospective, observational study of critically ill adult emergency department patients with sepsis or septic shock. Notably, inclusion criteria for the replication cohort included a SOFA score ≥ 4, compared to SOFA ≥ 2 for the derivation cohort, and therefore patients in the replication cohort had an overall higher severity of illness. All data and samples were obtained and processed in the first 24 h of sepsis recognition, as in the derivation set. Laboratory analyses were performed in an identical manner to the derivation cohort for this study. We first extracted the 15 features included in the signature and imputed the missing values using the features' median values [none of the patients had more than three (20%) missing features]. We then z-normalized the data using the means and scaling factors used in the initial dataset and processed feature outliers by capping their values to three standard deviations. Finally, we built the hierarchical clustering using the Spearman correlation and Ward method and extracted the first two clusters for analysis as above.

## Results

**Fig. 1:**
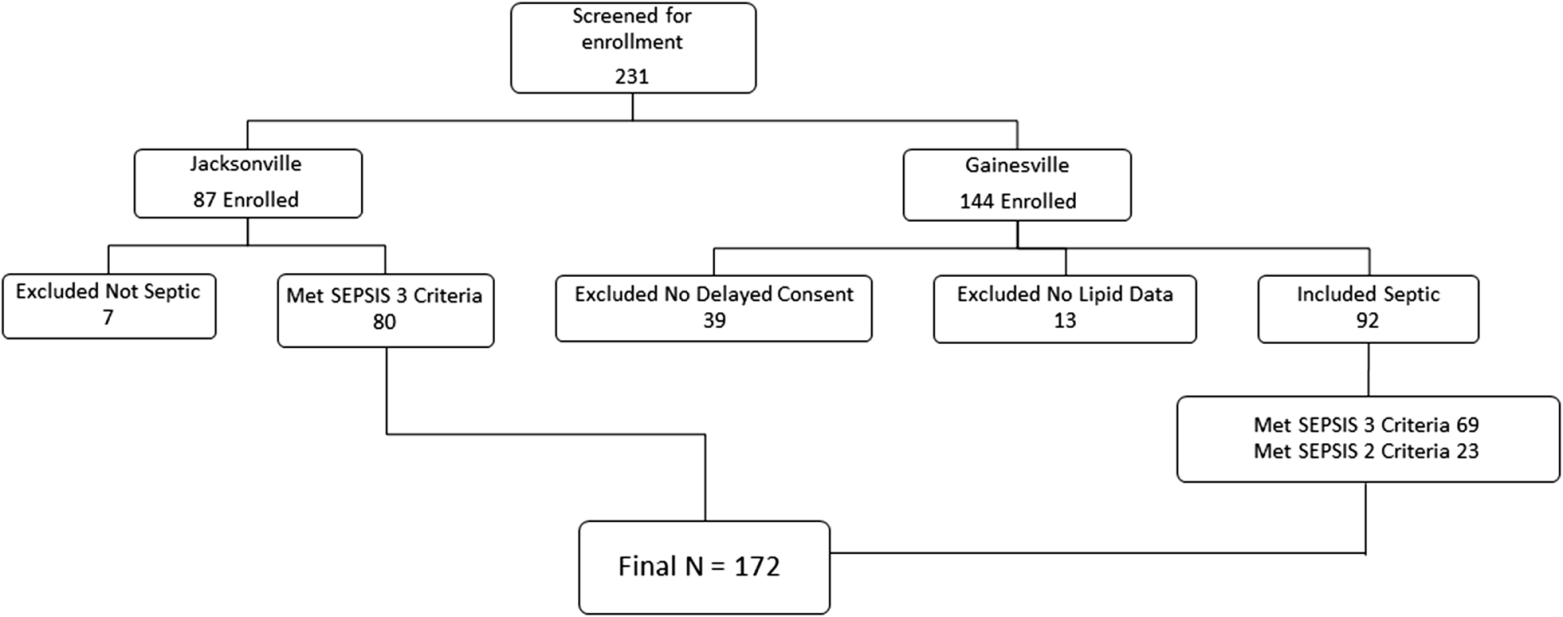
Enrollment flow chart for the derivation cohort

There were 172 septic patients enrolled in the study with at least one of two lipid measures upon enrollment (HII or PON-1), in addition to a lipid panel for cholesterol levels (Fig. 1—Enrollment Flow Diagram). Table 1 displays demographics, disease severity and comorbidity measures. The median age was 61 years (IQR 51–70); most patients were male, and most self-reported as White, followed by Black. After adjusting for multiple comparisons, comorbidity burden was similar across groups by Charlson Score. Enrollment SOFA and Apache II scores were significantly worse in early death and CCI patients. Early death patients had the highest SOFA scores at presentation (10, IQR 9–13), followed by CCI (7, IQR 5–10), and rapid recovery (4, IQR 2–6) (Table 1**)**. Most patients (67%, 114/172) had rapid recovery, while 23% (41/172) developed CCI, and 10% (17/172) had early death.

**Table 1:** Demographics, comorbidities, disease severity for the derivation cohort

Variable	All patients (N = 172)	Rapid Recovery (N = 114)		CCI (N = 41)	Early death (N = 17)	*p* Value^+^
*Demographics*						
Age, in years*	61 (51, 70)		60 (49, 69)	64 (56, 73)	63 (57, 74)	0.129^a^
Gender, male	99 (58)		63 (55)	25 (61)	11 (65)	0.671^b^
Race, White	123 (72)		78 (68)	33 (80)	12 (71)	0.364^c^
Black	47 (27)		35 (31)	7 (17)	5 (29)	
Other	2 (1)		1 (1)	1 (2)	0 (0)	
*Charlson comorbidity index*						
Charlson score*	3 (2, 5)		3 (1, 5)	4 (2, 6)	4 (3, 6)	0.019^a^
*Medications*						
Statin use	62 (36)		41 (37)	13 (32)	8 (47)	0.542^b^
*Initial biomarkers and organ failure*						
1st serum lactate (mmol/dL)*	2.1 (1.5, 3.2)		2 (1.5, 3)	1.70 (1.1, 2.9)	3.8 (2.8, 5)	** < 0.001**^**a**^
2nd serum lactate (mmol/dL)*	1.7 (1.1, 2.8)		1.5 (1.1, 2.4)	1.5 (1.1, 2.5)	4.1 (3.1, 8.0)	** < 0.001**^**a**^
Procalcitonin**	8.06 (2.3, 34.1)		13.3 (1.5, 27.1)	4.1 (1.8, 51.6)	7.6 (3.2, 100)	0.750^a^
Enrollment SOFA score*	5 (3, 8)		4 (2, 6)	7 (5, 10)	10 (9, 13)	** < 0.001**^**a**^
Apache II score*	15 (10, 20)		12 (8, 17)	19 (15, 25)	21 (18, 27)	** < 0.001**^**a**^

Bold values are significant after Bonferroni correction for multiple comparisons

Data is count (percentage), unless otherwise specified by *median (1st quartile, 3rd quartile); **n = 63; ^a^Wilcoxon rank-sum test; ^b^Pearson Chi-square test; ^c^Fisher’s exact test; ^+^after Bonferroni adjustment for multiple tests, significant if < 0.002 (0.05/47 tests). *Comparisons are bivariate between rapid recovery vs. CCI or early death*

Urinary tract infections were the most common source of sepsis, followed by intra-abdominal and pulmonary infections (Additional file 8: Table 1). Urinary tract infections were most frequent in rapid recovery patients while pulmonary infections were most common among CCI patients. There were no significant differences in intravenous fluid administration between groups, though there was a trend towards more fluids in early death patients at 24 h (Additional file 9: Table 2). There were also no significant differences in time to antibiotic administration. Mechanical ventilation duration and vasopressor usage were highest among early death and CCI patients compared to rapid recovery. Early death and CCI patients were more likely to be mechanically ventilated at enrollment. Poor disposition rates were higher for CCI than rapid recovery, and all mortality end points were higher for CCI (39% dead at 1 year). One-fourth (43/172) of all patients had died by one year.

### Lipid and lipoprotein biomarker associations

Enrollment total cholesterol levels (mg/dL) were lower in early death and CCI patients compared to rapid recovery (Table 2). HDL-C and LDL-C levels were lowest for early death patients, moderately low for CCI, and highest for rapid recovery. PON-1 enzymatic activity (*p* = 0.003) and ApoA-I levels (*p* < 0.0001) were higher in rapid recovery patients compared to early death and CCI. Inflammatory markers, including IL-8, IL-6, and IP-10 were also generally higher among early death and CCI patients compared to rapid recovery patients. Correlation matrices displaying Spearman’s correlations between lipid and lipoprotein markers (*x*-axis) and endothelial and inflammatory markers (*y*-axis) are displayed in Additional file 1: Fig. 1 (all patients) and Additional file 2: Fig. 2 (by outcome).

**Table 2:** Enrollment lipids and lipoproteins, endothelial, and inflammatory biomarkers by outcome

Variable	N	All patients	Rapid recovery	CCI	Early death	*p* Value^*,^^
*Lipid measures*						
Total cholesterol (mg/dL)	171	91 (67, 117)	100 (76, 120)	67 (56, 98)	71 (58, 117)	** < 0.001**
HDL-C (mg/dL)	171	19 (8, 30)	22 (10, 32)	14.5 (6, 20)	8 (5, 26)	**0.001**
LDL-C (mg/dL)	164	43.5 (27, 64)	50 (35, 67)	31 (21, 47)	25 (17, 59)	** < 0.001**
Triglycerides (mg/dL)	171	121 (78, 150)	120 (84, 149)	125 (75, 159)	118 (73, 143)	0.657
HDL inflammatory index	169	1.9 (1.2, 3.34)	1.7 (1.2, 3.56)	1.9 (1.3, 3.2)	2.6 (1.67, 3.1)	0.556
PON-1 activity (nmol/min/ml)	170	54 (29, 107)	66 (37, 115)	37 (25, 66)	43 (13, 83)	0.003
ApoA-1 (ng/mL)	152	1,100,000 (741,249, 1,500,000)	1,300,000 (919,877, 1,600,000)	819,148 (635,390, 1,000,000)	722,862 (564,171.5, 1,090,858.5)	** < 0.001**
*Endothelial markers*						
E-selectin (ng/mL)	133	68 (47, 143)	83 (51, 160)	55 (33, 78)	69 (52, 227)	0.025
ICAM (ng/mL)	139	426 (306, 617)	390 (291, 558)	448 (324, 669)	495 (439, 805)	0.055
*Inflammatory markers*						
MPO (µg/L)	150	203 (139, 334)	1889 (130, 323)	245 (161, 405)	277 (175, 459)	0.078
GRO (pg/mL)	152	1081 (537, 2000)	1100 (555, 1787)	1164 (533, 3519)	561 (324, 1189)	0.291
G-CSF (pg/mL)	164	334 (151, 1168)	329 (150, 1111)	335 (142, 799)	919 (181, 587)	0.256
GM-CSF (pg/mL)	164	12 (4, 41)	10 (4, 39)	13 (4, 42)	16 (7, 60)	0.244
IFNy (pg/mL)	164	28 (8, 62)	29 (8, 62)	25 (8, 53)	27 (11, 75)	0.815
IL-10 (pg/mL)	164	74 (31, 179)	64 (29, 161)	77 (52, 163)	291 (37, 1040)	0.082
IL-8 (pg/mL)	164	67 (31, 1501)	44 (23, 1034)	108 (57, 212)	180 (142, 812)	** < 0.001**
IL-6 (pg/mL)	164	176 (64, 520)	152 (52, 361)	259 (71, 919)	312 (152, 1986)	0.017
Il-12p70 (pg/mL)	164	12 (4, 27)	11 (4, 26)	12 (4, 22)	13 (4, 55)	0.726
IP-10 (pg/mL)	164	1066 (484, 2738)	981 (441, 2623)	900 (4878, 2192)	2235 (1638, 3807)	0.028
MCP-1 (pg/mL)	164	739 (460, 1732)	669 (441, 1397)	813 (523, 1779)	1246 (701, 3450)	0.071
MIP-1a (pg/mL)	164	8 (3, 18)	8 (3, 20)	6 (4, 12)	16 (8, 31)	0.055
TNFα (pg/mL)	164	70 (45, 138)	70 (40, 137)	63 (46, 118)	116 (63, 200)	0.103

Bold values are significant after Bonferroni correction for multiple comparisons

Data is median (1st quartile, 3rd quartile); *Wilcoxon rank-sum test; ^after Bonferroni adjustment for multiple tests, significant if < 0.002(0.05/47 tests). *Comparisons are bivariate between rapid recovery vs. CCI or early death*

The multivariable LASSO model was generated to predict CCI or early death using biomarkers and clinical features as predictors. After LASSO selection, the suggested model included: ApoA-I (log) (OR = 0.14, 95%CI 0.04, 0.47, *p* = 0.001), and LDL-C (OR = 0.98, 95%CI 0.95, 1.00, *p* = 0.062), mechanical ventilation use (OR = 17.94, 95%CI 5.61, 57.35, *p* < 0.0001), vasopressor use (OR = 3.96, 95%CI 1.46, 10.79, *p* = 0.007), and Charlson Comorbidity Score (OR = 1.24, 95%CI 1.02, 1.53, *p* = 0.035). ApoA-I (log) predicted reduced odds of CCI or early death, while mechanical ventilation use, vasopressor use and Charlson Comorbidity Score predicted increased odds of these outcomes. Although LDL-C levels variable did not reach statistical significance, the trend was towards predicting reduced odds of CCI or early death.

### Unsupervised clustering analysis

#### Derivation cohort

**Fig. 2:**
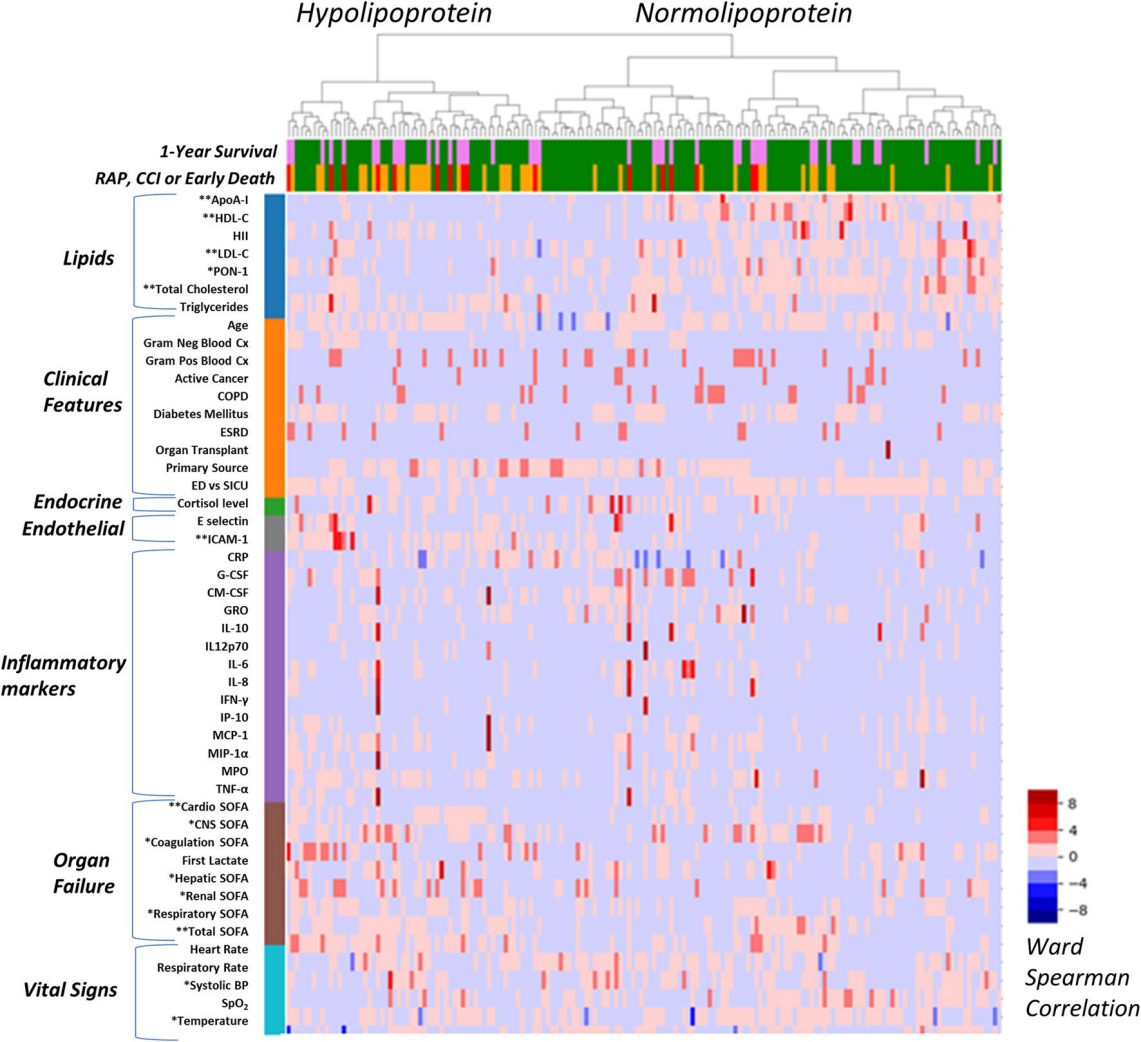
Heatmap demonstrating patient clusters (Hypolipoprotein vs. Normolipoprotein) on the *x*-axis with features (lipids, clinical variables, endocrine, endothelial or inflammatory biomarkers, organ failure severity, and vital signs) on the *y*-axis. For the *x*-axis (top), 1-year survival is presented with green representing 1-year survival, and pink representing 1-year death; for the *x*-axis (bottom), the primary outcomes of rapid recovery (RAP = green), chronic critical illness (CCI = orange), or early death (red) are presented

Unsupervised clustering yielded two clinically relevant clusters, referred to as the Hypolipoprotein cluster and the Normolipoprotein cluster (Fig. 2). The top five features that contributed to cluster discrimination were HDL-C, ApoA-I, total SOFA score, total cholesterol level, and ICAM-1 (Additional file 10: Table 3). Figure 2 illustrates the differences in biologic and clinical variables between clusters. The *y*-axis is organized by groups of features that contributed to cluster derivation, and the *x*-axis displays the outcomes of the patients in the clusters.

For the derivation cohort, patients in the Hypolipoprotein cluster were characterized by lower cholesterol levels, increased endothelial dysfunction (ICAM-1), and higher SOFA scores. Higher SOFA scores were largely driven by elevated cardiovascular SOFA scores, and increased incidence of shock and lower systolic blood pressure. Patients in Normolipoprotein cluster had higher cholesterol levels, less endothelial dysfunction, and lower SOFA scores. The Hypolipoprotein cluster was associated with significantly more lipid dysregulation, with lower levels of HDL-C, LDL-C, total cholesterol, ApoA-I, and PON compared to the Normolipoprotein cluster.

### Replication cohort

The replication cohort included 86 patients with sepsis or septic shock. Additional file 11: Supplemental Table 4 displays demographics and disease severity for the replication cohort. Table 3 displays data regarding significant features for the derivation and replication cohorts that were used in the unsupervised clustering signature. In the replication cohort, like the derivation cohort, there were significantly different values for HDL-C, ApoA-I, and ICAM-1 between the Hypolipoprotein and Normolipoprotein clusters. Hepatic SOFA scores were also comparatively higher in the Hypolipoprotein cluster. Lower PON-1 activity was also observed in the Hypolipoprotein cluster, though this was not significant. The heatmap for the replication cohort is displayed in Additional file 3: Fig. 3.

**Table 3:** Comparison of significant features from unsupervised clustering models between derivation and replication cohort

Lipid/oxidative marker at enrollment Median (25th, 75th)	Derivation cohort			Replication cohort		
	Hypolipoprotein cluster (*n* = 58)	Normolipoprotein cluster (*n* = 110)	*p* Value^+^	Hypolipoprotein cluster (*n* = 26)	Normolipoprotein cluster (*n* = 60)	*p* Value^^^
Total cholesterol (mg/dL)	67 (53, 84)	108 (81, 122)	** < 0.001**	87 (70, 102)	101 (82, 133)	0.047
HDL-C (mg/dL)	7 (5, 16)	26 (16, 33)	** < 0.001**	10 (5, 30)	34 (20, 46)	** < 0.001**
LDL-C (mg/dL)	30 (19, 44)	52 (36, 68)	**0.001**	41 (29, 68)	46 (35, 66)	0.267
PON-1 (mg/dL)	35.81 (22.4, 67.87)	65.99 (40.03, 114.6)	**0.001**	46.77 (23.59, 104.98)	89.42 (43.49, 128.59)	0.017
ApoA-I (ng/mL)	754,631 (583,580, 952,226)	1,400,000 (1,000,000, 1,625,000)	** < 0.001**	475785 (185360, 763480)	793130 (658255, 1030550)	** < 0.001**
ICAM-1 (ng/mL)	589.75 (455.82, 750.58)	346.502 (271.251, 485.513)	** < 0.001**	649.15 (516.80, 929.30)	351.92 (222.79, 384.97)	** < 0.001**
Cardiovascular SOFA	1 (1, 3)	1 (0, 1)	** < 0.001**	2 (0, 4)	1 (0, 3)	0.172
Neurologic SOFA	2 (0, 3)	0 (0, 2)	0.003	0 (0, 2)	2 (0, 3)	0.020
Coagulation SOFA	0 (0, 1)	0 (0, 1)	0.165	1 (0, 2)	0 (0, 1)	0.005
Hepatic SOFA	0 (0, 1)	0 (0, 0)	0.019	2 (1, 2)	0 (0, 0)	** < 0.001**
Renal SOFA	2 (0, 3)	1 (0, 2)	0.018	1 (0, 1)	1 (1, 3)	0.190
Respiratory SOFA	2 (0, 3)	0 (0, 2)	0.005	1 (0, 3)	2 (1, 3)	0.007
Systolic BP (mm Hg)	97 (88.25, 114)	113 (100.5, 126.75)	** < 0.001**	101 (92, 111)	108 (97, 123)	0.079
Temperature (˚F)	98.6 (98.1, 99.8)	99.6 (98.6, 101.3)	0.003	99.0 (97.6, 100.2)	99.4 (98.4, 100.5)	0.296
Total SOFA score	8 (6, 10)	4 (2, 6)	** < 0.001**	7 (5, 12)	7 (5, 10)	0.741

Bold values are significant after Bonferroni correction for multiple comparisons

Comparisons made using Wilcoxon rank-sum test; ^+^after Bonferroni adjustment for multiple tests, significant if < 0.001 (0.05/47 tests). ^^^For the replication cohort, after Bonferroni adjustment for multiple tests, significant if p-values < 0.003 (0.05/15 tests) were considered significant. *Comparisons are bivariate between Hypolipoprotein and Normolipoprotein Clusters separately for derivation and validation cohorts*

### Hypolipoprotein versus normolipoprotein outcomes comparisons

Table 4 displays outcomes data for the derivation and replication cohorts. In the derivation cohort, the Hypolipoprotein cluster accounted for the majority of patients who experienced CCI or early death and less than half of the patients who rapidly recovered. The Normolipoprotein cluster had more favorable outcomes after sepsis, this cluster was comprised of predominantly rapid recovery patients, with a small proportion of CCI or early death. In the replication cohort, though the proportion of rapid recovery patients across clusters was more evenly distributed, there was a much greater proportion of early death patients in the Hypolipoprotein cluster, and fewer CCI compared to the Normolipoprotein cluster. For both the derivation and replication cohorts, 28-day mortality rates were higher for the Hypolipoprotein cluster.

**Table 4:** Comparison of outcomes and 28-day mortality between derivation and replication cohort

Outcomes *N* (%)	Derivation cohort		Replication cohort	
	Hypolipoprotein Cluster (*n* = 58)	Normolipoprotein cluster (*n* = 110)	Hypolipoprotein cluster (*n* = 26)	Normolipoprotein cluster (*n* = 60)
Rapid recovery	26 (44.8)	87 (79.1)	12 (46.1)	33 (55.0)
Chronic critical illness	23 (39.7)	16 (14.5)	6 (23.1)	19 (31.7)
Early death	9 (15.5)	7 (6.4)	8 (30.8)	8 (13.3)
28-Day mortality	14 (20.7)	19 (14.6)	11 (42.3)	17 (28.3)

Data is count (percentage)

To compare the predictive ability of our unsupervised clustering signature for predicting rapid recovery vs. CCI or early death, Additional file 4–6: Figs. 4, 5, and 6 demonstrate the receiver operator characteristic (ROC) curves with areas under the curve (AUCs) for our unsupervised clustering signature compared with SOFA score and APACHE II. As can be seen here, our unsupervised clustering signature (AUC 0.75) was as good as the prediction provided by SOFA (0.755) and superior to that of APACHE II (70.5).

## Discussion

In this study, we demonstrated the predictive ability of lipids and lipoproteins for important clinical outcomes as previously defined by our group. We demonstrated that ApoA-I and LDL-C levels were predictive of the development of CCI or early death, in addition to mechanical ventilation, vasopressor use, and Charlson Comorbidity Score. Using unsupervised clustering, we were then able to phenotype sepsis patients into Hypolipoprotein and Normolipoprotein phenotypes, with the Hypolipoprotein phenotype being characterized by lower lipoprotein levels (lower HDL-C and ApoA-I), and increased endothelial dysfunction (ICAM-1) as well as higher SOFA scores. These findings were validated in an independent replication cohort of ED patients with sepsis or septic shock.

Our finding that ApoA-I and LDL-C, in addition to mechanical ventilation use, vasopressor use, and Charlson Score being predictive of CCI and early death, is not entirely new. Pavlou et al. as well as Zou et al. have previously shown that decreasing ApoA-I levels are associated with poor outcomes in sepsis [51, 52]. And others have shown the prognostic ability of LDL-C [53]. In this study, we used a diverse cohort of sepsis patients that contained both community-acquired and hospital-acquired sepsis from two settings at two hospitals.

Using our unsupervised clustering approach, we identified, and verified in an independent replication cohort, a Hypolipoprotein phenotype of sepsis that is represented by lower levels of key lipoproteins, and is associated with increased endothelial dysfunction, organ failure, and poorer outcomes. In the derivation cohort, the Hypolipoprotein phenotype had significantly lower total cholesterol, HDL-C, LDL-C, PON-1, ApoA-I, and higher ICAM-1, indicative of more severe endothelial dysfunction. The low levels of PON-1 and ApoA-I also indicate the reduced antioxidant and anti-inflammatory capacity of patients in the Hypolipoprotein phenotype. Notably, the derivation cohort had higher cardiovascular SOFA scores, which contributed to overall higher severity of organ failure. Similarly, the replication cohort also had lower levels of HDL-C, ApoA-I, and higher ICAM-1. Additionally, the Hypolipoprotein phenotype in the replication cohort had significantly higher hepatic SOFA scores compared to the Normolipoprotein phenotype. One reason for the observed difference in hepatic dysfunction between derivation and replication cohorts is that severe hepatic failure was an exclusion for the derivation cohort, but not for the replication cohort. The exclusion of patients with severe hepatic failure and lipoprotein metabolic disorders, may have resulted in the exclusion of patients prone to low or abnormal lipoprotein levels.

Though our sample size may have limited our ability to detect more nuanced phenotypes in these analyses, it suggests that lipoprotein pathobiology may contribute to sepsis outcome heterogeneity. In our independent replication cohort, we were able to validate our two phenotypes and their associated outcomes. Further, combined with our multivariable regression results, our clustering results provide additional evidence supporting the relationship between lipoprotein dysregulation and morbid outcomes from sepsis.

Our biomarker correlation matrices provide insight into the biological underpinnings of aforementioned findings. The correlation matrices demonstrated strong, inverse relationships between HDL-C, ApoA-I, LDL-C, total cholesterol, and PON-1 with endothelial dysfunction markers but a positive correlation with triglycerides. This may indicate increased endothelial dysfunction as lipid levels and antioxidant capacity decrease in sepsis due to degradation, consumption, or transfer of lipids or lipoproteins to other moieties. This finding is supported by prior studies that have shown that HDL-C has potent endothelial protective effects, and may reflect loss of HDL’s endothelial protective effects in sepsis [24, 25, 54, 55]. HII was also shown to be positively correlated with MPO and MCP-1, both of which are measures of oxidation and inflammation, and likely reflects increased oxidized lipids in sepsis [56, 57]. In addition, the inverse association of nearly all the cholesterol measures with the inflammatory markers IL-6 and IL-8, as well as the correlation between HDL-C and ApoA-I with IL-10, GM-CSF, MPO, and TNFα is notable, as it demonstrates that HDL-C and ApoA-I may be more affected by the oxidizing effects of MPO and other inflammatory markers (TNF, IL-10) than other lipids. Finally, in addition to the reduction in cholesterol levels that has been previously described [53, 58, 59], our study demonstrated that PON-1 and ApoA-I levels were significantly higher in patients who rapidly recovered compared to CCI or early death, indicating reduced lipoprotein antioxidant capacity in patients with poor outcomes. Unsurprisingly, measures of endothelial dysfunction (E-selectin and ICAM-1) and inflammatory markers (IL-8, IL-6, and IP-10) also demonstrated significant differences between rapid recovery, CCI, and early death groups. IL-6, which stimulates acute phase proteins including serum amyloid A, and has been previously demonstrated to displace up to 45% of ApoA-I from circulating HDL-C in sepsis was also elevated, and may contribute to Dys-HDL pathogenesis [37, 60].

In summary, the results of our unsupervised clustering analysis and biomarker profiling suggest that reduced levels of protective lipids (HDL-C, ApoA-I, and LDL-C) are associated with increased endothelial dysfunction (E-selectin, ICAM-1) and worse outcomes. The reduction in these protective lipoproteins is also associated with increased inflammatory markers, HDL oxidation (elevated HII), and reduced PON-1 activity.

### Limitations

This study had several limitations. First, with only 172 patients included in the final analysis, this study was a relatively small. However, concordant findings reported from two independent health centers with very different demographics (medical vs surgical sepsis; community vs. hospital-acquired sepsis; and small city vs. large inner-city urban) instill confidence in our analyses. In addition, the findings of our unsupervised clustering approach were validated in a second independent replication cohort. Second, although we used rigorous statistical approaches, the reported findings need to be validated by investigators at other settings, as both our derivation and independent replication cohorts were from the same institutions. Third, our biomarker findings are correlative and do not adjust for other covariates, and as such, should be interpreted with caution. Fourth, we did not exclude patients on propofol infusions, and though rarely used for sedation in ventilated septic patients at our institution, this could have influenced lipid levels. With regards to the machine learning analysis, the sample size limited our ability to detect additional, more nuanced subgroups. Given that the data included a substantial number of lipid-related variables, lipid data may have contributed disproportionately to cluster derivation. Thus, these findings should be interpreted as hypothesis generating for the relationship between lipid dysregulation and sepsis heterogeneity.

## Conclusion

Here, we demonstrated that a Hypolipoprotein phenotype of sepsis, represented by lower levels of HDL-C, ApoA-I, and reduced PON-1 activity is associated with increased endothelial dysfunction, organ failure, and poorer outcomes compared to a Normolipoprotein phenotype. We showed that HDL-C and ApoA-I specifically have important associations with markers of endothelial dysfunction and have strong discriminative ability for poor versus favorable outcomes in a diverse sepsis cohort using classic and machine learning analytic approaches. Future studies should focus on the mechanisms by which lipids and lipoproteins influence sepsis heterogeneity and outcomes.

## Supplementary Information

**Additional file 1: Figure 1.** Correlation matrix of biomarkers for the whole cohort. Vertical representation (y-axis) of endothelial (gray) and inflammatory (purple) biomarkers with lipid measures (blue) on the horizontal (x-axis). All correlations were performed using Spearman’s correlations for non-parametric data. Biomarkers, Y axis: ICAM (human intercellular adhesion molecule-1), G-CSF (granulocyte colony stimulating factor), GM-CSF (granulocyte macrophage stimulating factor), GRO (growth related oncogene, IL-10 (interleukin 10), IL-12p70, IL-6, IL-8, IFN-γ (interferon gamma), IP-10 (interferon gamma-induced protein), MCP-1 (monocyte chemotactic protein-1), MIP-1 α (macrophage inflammatory protein-1α), MPO (myeloperoxidase), tumor necrosis factor alpha (TNF-α); X axis: TG (triglycerides), TC (total cholesterol), PON-1 (paraoxonase-1), LDL (low density lipoprotein cholesterol), HII (HDL inflammatory index), HDL (high density lipoprotein cholesterol), ApoA-I (apolipoprotein A-I).

**Additional file 2: Figure 2.** Correlation matrix of biomarkers at the time of enrollment by outcome. Vertical representation (y-axis) of endothelial (gray) and inflammatory (purple) biomarkers with lipid measures (blue) on the horizontal (x-axis). All correlations were performed using Spearman’s correlations for non-parametric data.

**Additional file 3: Figure 3.** Heatmap demonstrating patient clusters (Hypolipoprotein vs. Normolipoprotein) on the x-axis, with the 15 significant features identified in the derivation cohort represented on the y-axis.

**Additional file 4: Figure 4.** ROC Curve showing lipoprotein signature prediction of Rapid Recovery vs. Chronic Critical Illness or Early Death.

**Additional file 5: Figure 5.** ROC curve of SOFA Score prediction of Rapid Recovery vs. Chronic Critical Illness or Early Death.

**Additional file 6: Figure 6.** ROC curve of APACHE II Score prediction of Rapid Recovery vs. Chronic Critical Illness or Early Death.

**Additional file 7: Figure 7.** The Calinski-Harabasz score as a function of the number of clusters obtained using the KMean algorithm implemented in the scikit-learn Python library (version 0.24.2). The Calinski-Harabasz score captures how similar members of each cluster are (compactness) as well as separation between clusters.

**Additional file 8: Supplemental Table 1.** Presenting features and infectious source for derivation cohort.

**Additional file 9: Supplemental Table 2.** Clinical management and outcomes for the derivation cohort.

**Additional file 10: Supplemental Table 3.** Top features contributing to cluster discrimination between Hypolipoprotein and Normolipoprotein Cluster.

**Additional file 11: Supplemental Table 4.** Demographics and disease severity for the replication cohort.

## Data Availability

Supporting data from this study can be obtained by emailing the corresponding author Dr. Faheem W. Guirgis, MD.

## References

[1] Rudd KE, Johnson SC, Agesa KM (2020). Global, regional, and national sepsis incidence and mortality, 1990–2017: analysis for the Global Burden of Disease Study. Lancet.

[2] Gaieski DF, Edwards JM, Kallan MJ, Carr BG (2013). Benchmarking the incidence and mortality of severe sepsis in the united states. Crit Care Med.

[3] Singer M, Deutschman CS, Seymour C (2016). The third international consensus definitions for sepsis and septic shock (Sepsis-3). JAMA.

[4] Kumar G, Kumar N, Taneja A, Al E (2011). Nationwide trends of severe sepsis in the 21st century (2000–2007). Chest.

[5] Guirgis FW, Brakenridge S, Sutchu S (2016). The long-term burden of severe sepsis and septic shock: Sepsis recidivism and organ dysfunction. J Trauma Acute Care Surg.

[6] Gardner AK, Ghita GL, Wang Z (2019). The development of chronic critical illness determines physical function, quality of life, and long-term survival among early survivors of sepsis in surgical ICUs*. Crit Care Med.

[7] Gentile LF, Cuenca AG, Efron PA (2012). Persistent inflammation and immunosuppression: a common syndrome and new horizon for surgical intensive care. J Trauma Acute Care Surg.

[8] Mira JC, Gentile LF, Mathias BJ (2016). Sepsis pathophysiology, chronic critical illness, and persistent inflammation-immunosuppression and catabolism syndrome. Crit Care Med.

[9] Morin EE, Guo L, Schwendeman A, Li X-A (2015). HDL in sepsis—risk factor and therapeutic approach. Front Pharmacol.

[10] Walley KR (2016). Role of lipoproteins and proprotein convertase subtilisin/kexin type 9 in endotoxin clearance in sepsis. Curr Opin Crit Care.

[11] Khovidhunkit W, Kim M-S, Memon RA (2004). Effects of infection and inflammation on lipid and lipoprotein metabolism: mechanisms and consequences to the host. J Lipid Res.

[12] İnal V, Yamanel L, Taşkın G, Tapan S, Cömert B (2015). Paraoxonase 1 activity and survival in sepsis patients. Balkan Med J.

[13] Liao XL, Lou B, Ma J, Wu MP (2005). Neutrophils activation can be diminished by apolipoprotein A-I. Life Sci.

[14] Murphy AJ, Woollard KJ, Hoang A (2008). High-density lipoprotein reduces the human monocyte inflammatory response. Arterioscler Thromb Vasc Biol.

[15] Murphy AJ, Woollard KJ, Suhartoyo A (2011). Neutrophil activation is attenuated by high-density lipoprotein and apolipoprotein A-I in in vitro and in vivo models of inflammation. Arterioscler Thromb Vasc Biol.

[16] De Nardo D, Labzin LI, Kono H (2014). High-density lipoprotein mediates anti-inflammatory reprogramming of macrophages via the transcriptional regulator ATF3. Nat Immunol.

[17] Catapano AL, Pirillo A, Bonacina F, Norata GD. HDL in innate and adaptive immunity. *Cardiovasc Res*. 2014. cvu150 [pii]. 10.1093/cvr/cvu150 24935428

[18] Beutler B, Hoebe K, Du X, Ulevitch RJ (2003). How we detect microbes and respond to them: the Toll-like receptors and their transducers. J Leukoc Biol.

[19] Parrillo JE (1993). Pathogenetic mechanisms of septic shock. N Engl J Med.

[20] Kitchens RL, Wolfbauer G, Albers JJ, Munford RS (1999). Plasma lipoproteins promote the release of bacterial lipopolysaccharide from the monocyte cell surface. J Biol Chem.

[21] Topchiy E, Cirstea M, Kong HJ, et al. Lipopolysaccharide is cleared from the circulation by hepatocytes via the low density lipoprotein receptor. Tancevski I, ed. PLoS ONE. 2016;11(5):e0155030. 10.1371/journal.pone.0155030. 10.1371/journal.pone.0155030 PMC4865154 27171436

[22] Boyd JH, Fjell CD, Russell JA, Sirounis D, Cirstea MS, Walley KR (2016). Increased plasma PCSK9 levels are associated with reduced endotoxin clearance and the development of acute organ failures during sepsis. J Innate Immun.

[23] Walley KR, Thain KR, Russell JA (2014). PCSK9 is a critical regulator of the innate immune response and septic shock outcome. Sci Transl Med..

[24] Celermajer DS (1997). Endothelial dysfunction: does it matter? Is it reversible?. J Am Coll Cardiol.

[25] Norata GD, Catapano AL (2005). Molecular mechanisms responsible for the antiinflammatory and protective effect of HDL on the endothelium. Vasc Health Risk Manag.

[26] Barlage S, Gnewuch C, Liebisch G (2009). Changes in HDL-associated apolipoproteins relate to mortality in human sepsis and correlate to monocyte and platelet activation. Intensive Care Med.

[27] Cirstea M, Walley KR, Russell JA, Brunham LR, Genga KR, Boyd JH (2017). Decreased high-density lipoprotein cholesterol level is an early prognostic marker for organ dysfunction and death in patients with suspected sepsis. J Crit Care.

[28] Lekkou A, Mouzaki A, Siagris D, Ravani I, Gogos CA (2014). Serum lipid profile, cytokine production, and clinical outcome in patients with severe sepsis. J Crit Care.

[29] Tanaka S, Stern J, Bouzid D (2021). Relationship between lipoprotein concentrations and short-term and 1-year mortality in intensive care unit septic patients: results from the HIGHSEPS study. Ann Intensive Care.

[30] Pirillo A, Catapano AL, Norata GD. HDL in infectious diseases and sepsis. In: Handbook of experimental pharmacology; 2015. 10.1007/978-3-319-09665-0_15 10.1007/978-3-319-09665-0_15 25522999

[31] Trinder M, Walley KR, Boyd JH, Brunham LR (2020). Causal inference for genetically determined levels of high-density lipoprotein cholesterol and risk of infectious disease. Arterioscler Thromb Vasc Biol.

[32] Trinder M, Genga KR, Kong HJ (2019). Cholesteryl ester transfer protein influences high-density lipoprotein levels and survival in sepsis. Am J Respir Crit Care Med.

[33] Guirgis FW, Dodani S, Moldawer L (2017). Exploring the predictive ability of dysfunctional high density lipoprotein for adverse outcomes in emergency department patients with sepsis. Shock.

[34] Guirgis FW, Dodani S, Leeuwenburgh C (2018). HDL inflammatory index correlates with and predicts severity of organ failure in patients with sepsis and septic shock. PLoS ONE.

[35] Guirgis FW, Leeuwenburgh C, Grijalva V (2018). HDL cholesterol efflux is impaired in older patients with early sepsis: a subanalysis of a prospective pilot study. Shock.

[36] Pirillo A, Catapano AL, Norata GD (2015). HDL in infectious diseases and sepsis. Handb Exp Pharmacol.

[37] van Leeuwen HJ, Heezius ECJM, Dallinga GM, van Strijp JAG, Verhoef J, van Kessel KPM (2003). Lipoprotein metabolism in patients with severe sepsis. Crit Care Med.

[38] Tanaka S, Diallo D, Delbosc S (2019). High-density lipoprotein (HDL) particle size and concentration changes in septic shock patients. Ann Intensive Care.

[39] Loftus TJ, Mira JC, Ozrazgat-Baslanti T (2017). Sepsis and Critical Illness Research Center investigators: protocols and standard operating procedures for a prospective cohort study of sepsis in critically ill surgical patients. BMJ Open.

[40] von Elm E, Altman DG, Egger M, Pocock SJ, Gøtzsche PC, Vandenbroucke JP (2008). The strengthening the reporting of observational studies in epidemiology (STROBE) statement: guidelines for reporting observational studies. J Clin Epidemiol.

[41] Brakenridge SC, Lysak N, Ghita G (2018). Comparison of sepsis-2 and sepsis-3 clinical criteria in critically ill patients: Is there any impact on discrimination of immunophenotype and clinical outcomes?. Shock.

[42] Friedewald WT, Levy RI, Fredrickson DS (1972). Estimation of the concentration of low-density lipoprotein cholesterol in plasma, without use of the preparative ultracentrifuge. Clin Chem.

[43] Guirgis FW, Leeuwenburgh C, Grijalva V (2017). HDL cholesterol efflux is impaired in older patients with early sepsis. Shock.

[44] Harris PA, Taylor R, Thielke R, Payne J, Gonzalez N, Conde JG (2009). Research electronic data capture (REDCap)—a metadata-driven methodology and workflow process for providing translational research informatics support. J Biomed Inform.

[45] Charlson ME, Pompei P, Ales KL, MacKenzie CR (1987). A new method of classifying prognostic comorbidity in longitudinal studies: development and validation. J Chronic Dis.

[46] Brakenridge SC, Efron PA, Cox MC (2019). Current epidemiology of surgical sepsis: discordance between inpatient mortality and 1-year outcomes. Ann Surg.

[47] Tibshirani R (1996). Regression shrinkage and selection via the lasso. J R Stat Soc Ser B.

[48] Statistics corner: A guide to appropriate use of correlation coefficient in medical research—PubMed. https://pubmed.ncbi.nlm.nih.gov/23638278/. Accessed 20 Aug 2021. PMC3576830 23638278

[49] Edelbrock C, McLaughlin B (1980). Hierarchical cluster analysis using intraclass correlations: a mixture model study. Multivariate Behav Res..

[50] Guirgis FW, Dodani S, Leeuwenburgh C, et al. HDL inflammatory index correlates with and predicts severity of organ failure in patients with sepsis and septic shock. Calabresi L, ed. PLoS ONE. 2018;13(9):e0203813. 10.1371/journal.pone.0203813. 10.1371/journal.pone.0203813 PMC6138388 30216360

[51] Pavlou E, Makris K, Palaiologou A (2008). Decreased apolipoprotein A1 levels correlate with sepsis and adverse outcome among ICU patients. Crit Care.

[52] Zou G, He J, Ren B, Xu F, Xu G, Zhang W (2016). The delta high-density lipoprotein cholesterol ratio: a novel parameter for gram-negative sepsis. Springerplus.

[53] Walley KR, Boyd JH, Kong HJ, Russell JA (2018). Low low-density lipoprotein levels are associated with, but do not causally contribute to, increased mortality in sepsis. Crit Care Med.

[54] Landmesser U, Hornig B, Drexler H. Endothelial function: a critical determinant in atherosclerosis? *Circulation*. 2004;109:II27–I33. 10.1161/01.CIR.0000129501.88485.1f 10.1161/01.CIR.0000129501.88485.1f 15173060

[55] Spirig R, Schaub A, Kropf A, Miescher S, Spycher MO, Rieben R (2013). Reconstituted high-density lipoprotein modulates activation of human leukocytes. PLoS ONE.

[56] Charles-Schoeman C, Lee YY, Grijalva V (2012). Cholesterol efflux by high density lipoproteins is impaired in patients with active rheumatoid arthritis. Ann Rheum Dis.

[57] Yunoki K, Naruko T, Inaba M (2013). Gender-specific correlation between plasma myeloperoxidase levels and serum high-density lipoprotein-associated paraoxonase-1 levels in patients with stable and unstable coronary artery disease. Atherosclerosis.

[58] Chien Y-F, Chen C-Y, Hsu C-L, Chen K-Y, Yu C-J (2015). Decreased serum level of lipoprotein cholesterol is a poor prognostic factor for patients with severe community-acquired pneumonia that required intensive care unit admission. J Crit Care.

[59] Chien J-Y, Jerng J-S, Yu C-J, Yang P-C (2005). Low serum level of high-density lipoprotein cholesterol is a poor prognostic factor for severe sepsis. Crit Care Med.

[60] Tanaka T, Narazaki M, Kishimoto T (2014). Il-6 in inflammation, immunity, and disease. Cold Spring Harb Perspect Biol.

