# Supplementary figures and images for: A hypolipoprotein sepsis phenotype indicates reduced lipoprotein antioxidant capacity, increased endothelial dysfunction and organ failure, and worse clinical outcomes
Source: Crit Care. 2021 Sep 17;25:341. doi: 10.1186/s13054-021-03757-5 (PMC8447561; doi:10.1186/s13054-021-03757-5)

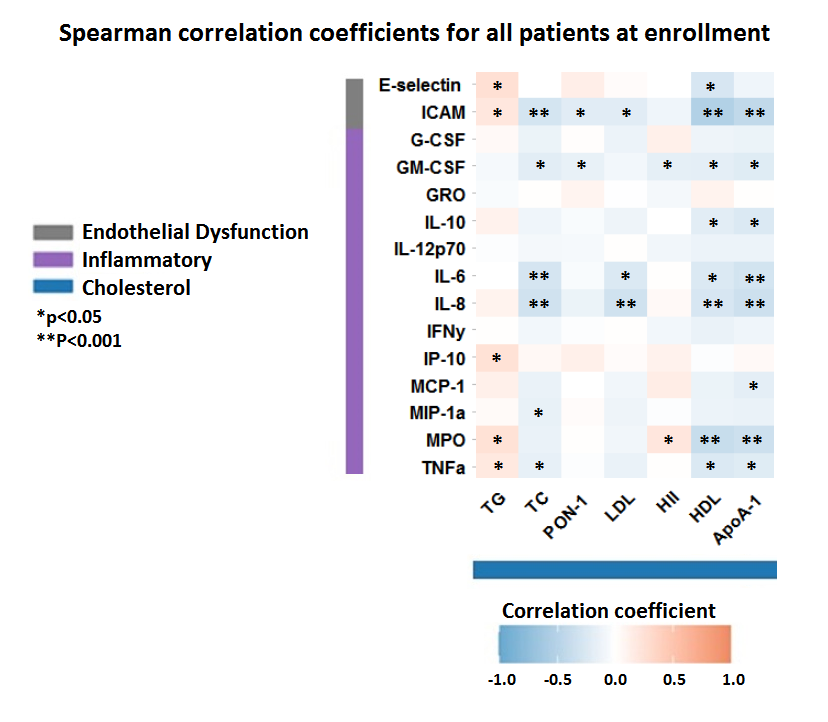

Supplement: Supplementary file 1 — Additional file 1: Figure 1. Correlation matrix of biomarkers for the whole cohort. Vertical representation (y-axis) of endothelial (gray) and inflammatory (purple) biomarkers with lipid measures (blue) on the horizontal (x-axis). All correlations were performed using Spearman’s correlations for non-parametric data. Biomarkers, Y axis: ICAM (human intercellular adhesion molecule-1), G-CSF (granulocyte colony stimulating factor), GM-CSF (granulocyte macrophage stimulating factor), GRO (growth related oncogene, IL-10 (interleukin 10), IL-12p70, IL-6, IL-8, IFN-γ (interferon gamma), IP-10 (interferon gamma-induced protein), MCP-1 (monocyte chemotactic protein-1), MIP-1 α (macrophage inflammatory protein-1α), MPO (myeloperoxidase), tumor necrosis factor alpha (TNF-α); X axis: TG (triglycerides), TC (total cholesterol), PON-1 (paraoxonase-1), LDL (low density lipoprotein cholesterol), HII (HDL inflammatory index), HDL (high density lipoprotein cholesterol), ApoA-I (apolipoprotein A-I). [file 13054_2021_3757_MOESM1_ESM.tif]

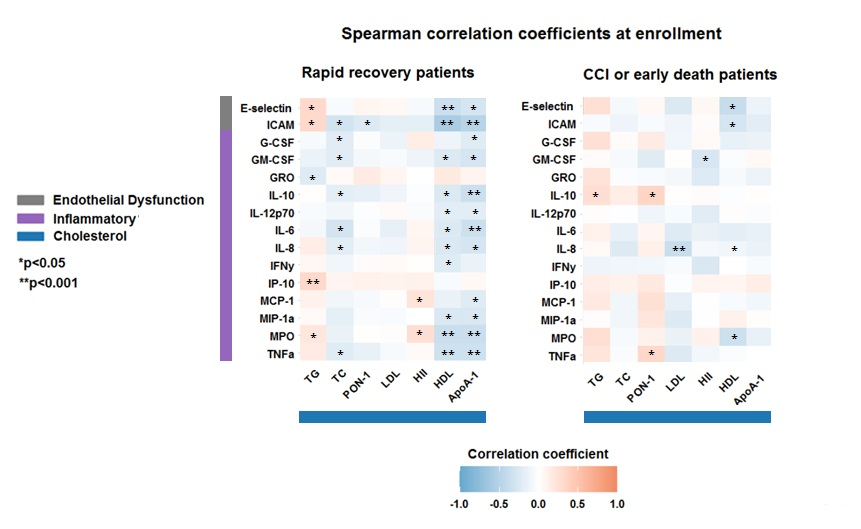

Supplement: Supplementary file 2 — Additional file 2: Figure 2. Correlation matrix of biomarkers at the time of enrollment by outcome. Vertical representation (y-axis) of endothelial (gray) and inflammatory (purple) biomarkers with lipid measures (blue) on the horizontal (x-axis). All correlations were performed using Spearman’s correlations for non-parametric data. [file 13054_2021_3757_MOESM2_ESM.tif]

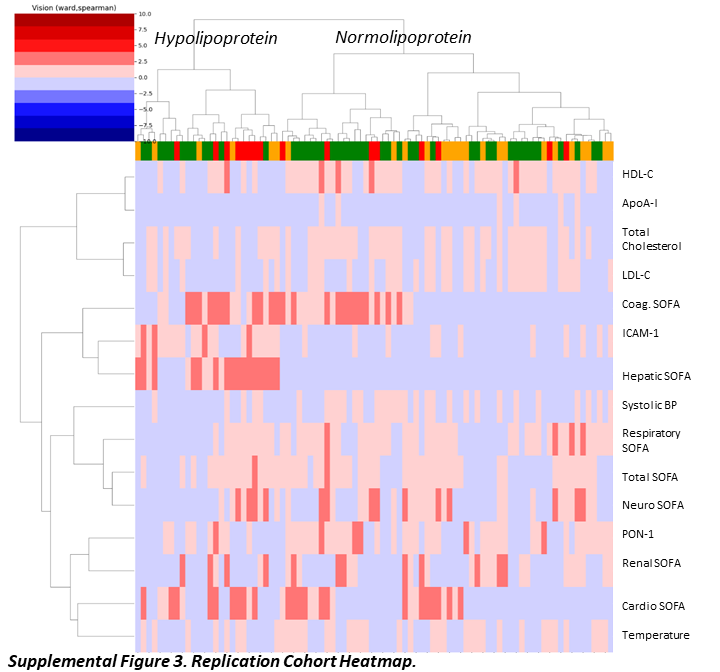

Supplement: Supplementary file 3 — Additional file 3: Figure 3. Heatmap demonstrating patient clusters (Hypolipoprotein vs. Normolipoprotein) on the x-axis, with the 15 significant features identified in the derivation cohort represented on the y-axis. [file 13054_2021_3757_MOESM3_ESM.tif]

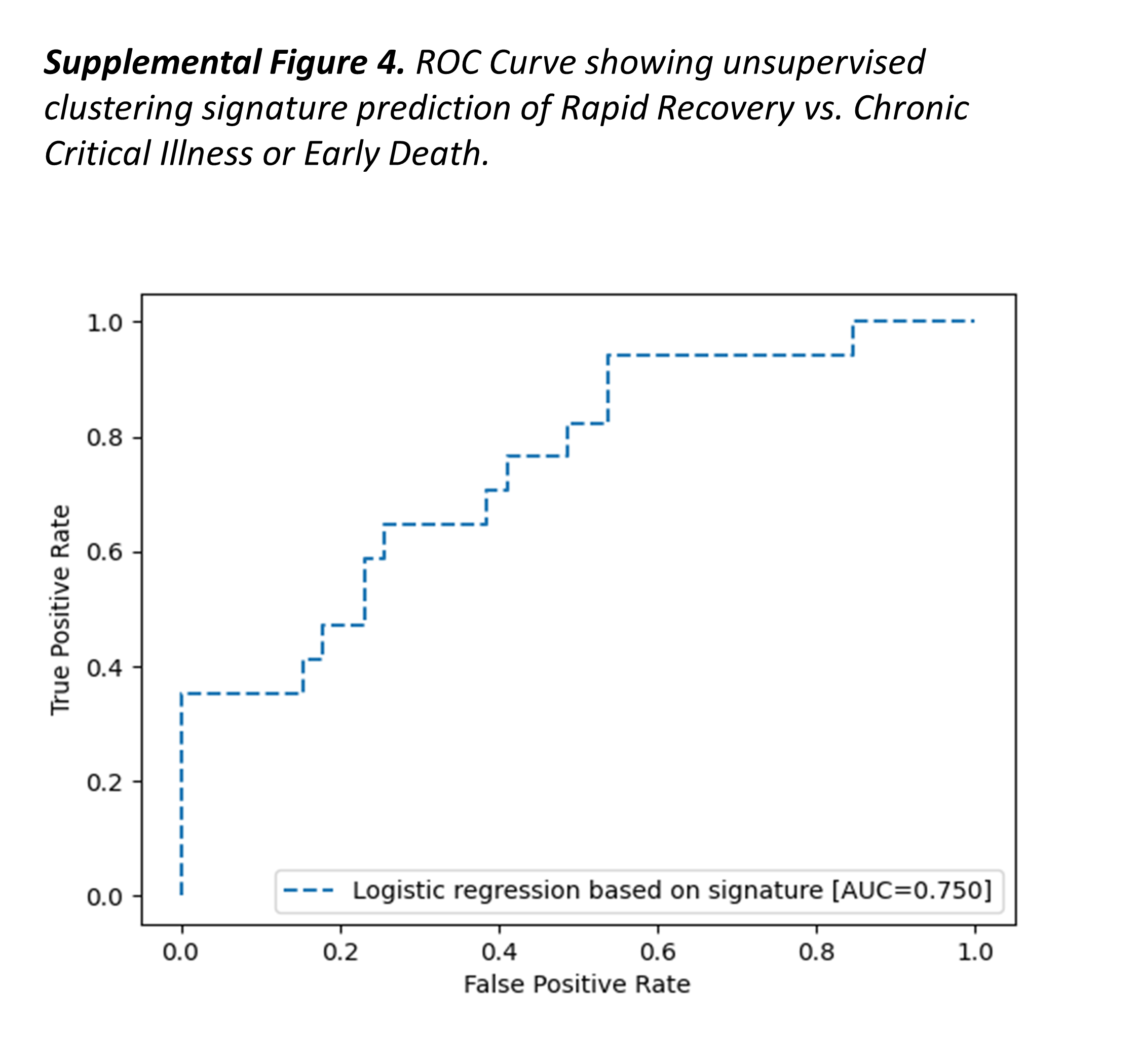

Supplement: Supplementary file 4 — Additional file 4: Figure 4. ROC Curve showing lipoprotein signature prediction of Rapid Recovery vs. Chronic Critical Illness or Early Death. [file 13054_2021_3757_MOESM4_ESM.tif]

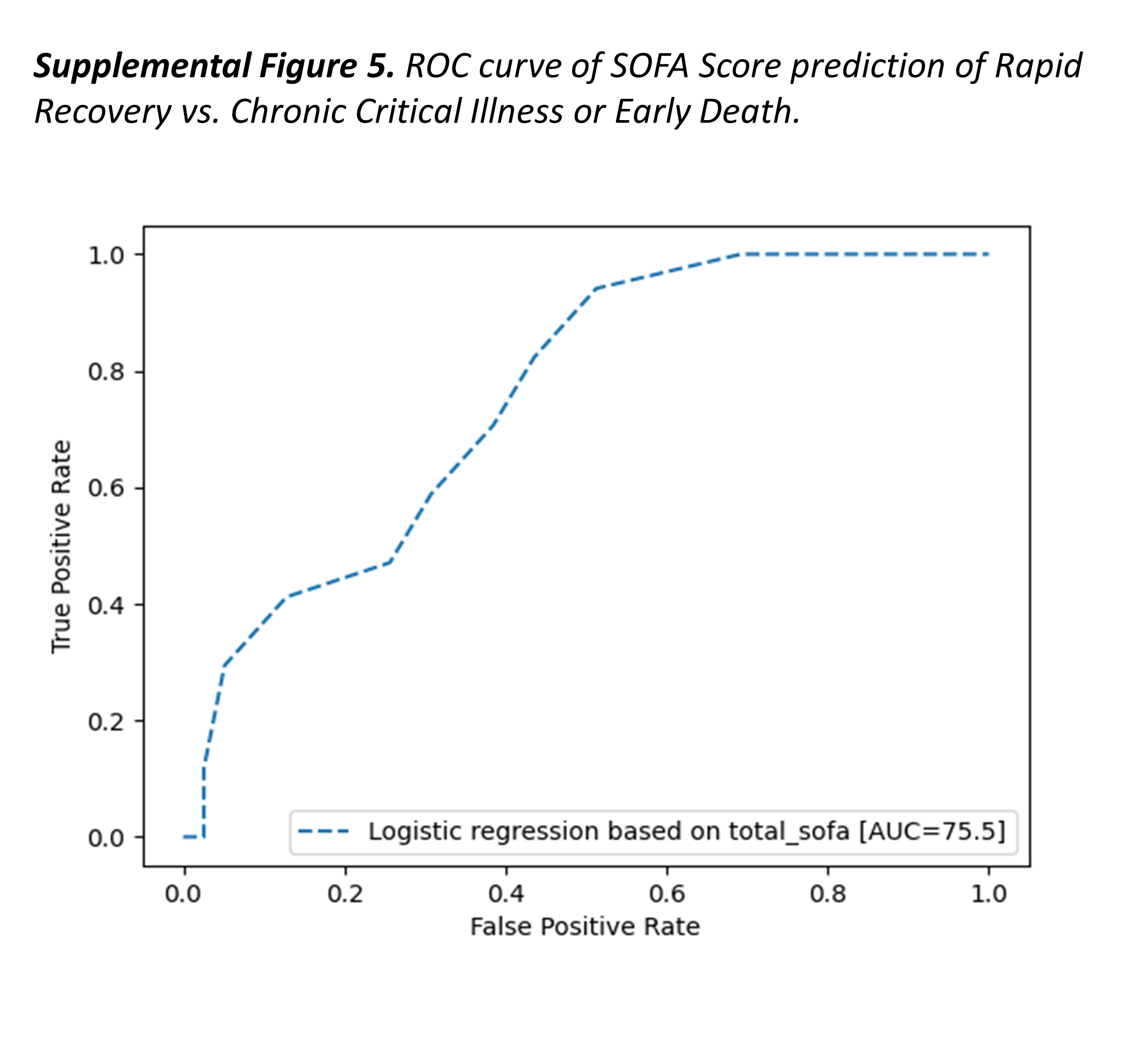

Supplement: Supplementary file 5 — Additional file 5: Figure 5. ROC curve of SOFA Score prediction of Rapid Recovery vs. Chronic Critical Illness or Early Death. [file 13054_2021_3757_MOESM5_ESM.tif]

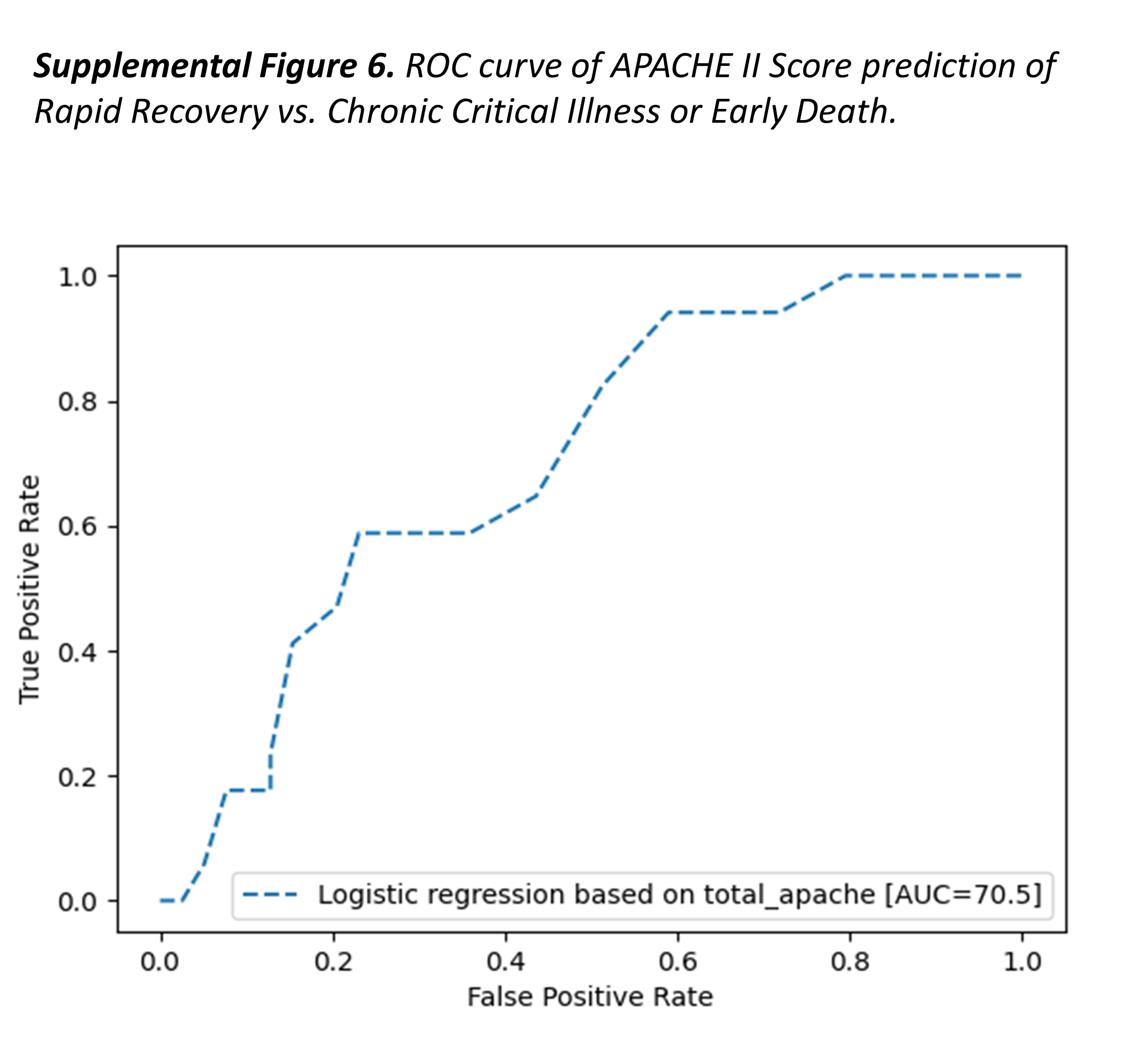

Supplement: Supplementary file 6 — Additional file 6: Figure 6. ROC curve of APACHE II Score prediction of Rapid Recovery vs. Chronic Critical Illness or Early Death. [file 13054_2021_3757_MOESM6_ESM.tif]

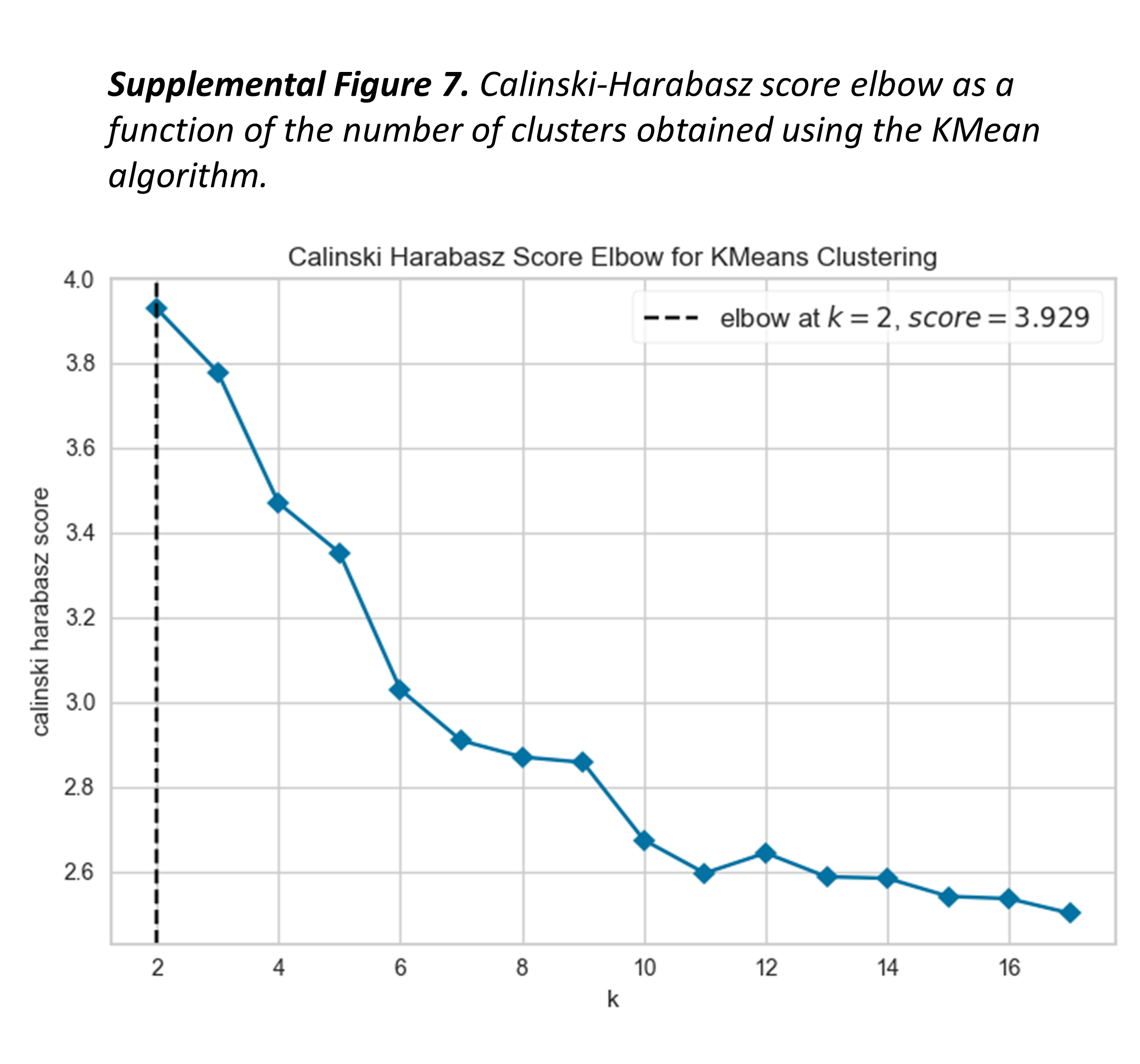

Supplement: Supplementary file 7 — Additional file 7: Figure 7. The Calinski-Harabasz score as a function of the number of clusters obtained using the KMean algorithm implemented in the scikit-learn Python library (version 0.24.2). The Calinski-Harabasz score captures how similar members of each cluster are (compactness) as well as separation between clusters. [file 13054_2021_3757_MOESM7_ESM.tif]
