# Supplementary material for: A hypolipoprotein sepsis phenotype indicates reduced lipoprotein antioxidant capacity, increased endothelial dysfunction and organ failure, and worse clinical outcomes
Source: Crit Care. 2021 Sep 17;25:341. doi: 10.1186/s13054-021-03757-5 (PMC8447561; doi:10.1186/s13054-021-03757-5)
Supplement: Supplementary file 8 — Additional file 8: Supplemental Table 1. Presenting features and infectious source for derivation cohort. [file 13054_2021_3757_MOESM8_ESM.docx]

**Supplemental Table 1. Presenting features and infectious source for derivation cohort.**

| **Variable** | **All patients**  **(N = 172)** | **Rapid Recovery**  **(N=114)** | **CCI**  **(N=41)** | **Early Death**  **(N=17)** |
| --- | --- | --- | --- | --- |
| **Presenting Clinical Features*** | | | | |
| Systolic blood pressure (mm Hg) | 110 (93, 131) | 114 (96, 134) | 108 (92, 117) | 107 (89, 131)  95 (87, 111)  20 (18, 22)  98.4 (97.3, 98.9) |
| Heart rate (beats/min) | 110 (95.5, 124) | 110 (97, 125) | 111 (98, 124) |  |
| Respiratory rate (breaths/min) | 20 (18, 27) | 20 (18, 26) | 21 (18, 29) |  |
| Temperature (®F) | 99.2 (98.1, 101.1) | 99.3 (98.2, 101.5) | 99.5 (97.7, 101.5) |  |
| Oxygen saturation (%) | 97 (94, 99) | 96.5 (94, 99) | 97 (95, 99) | 97 (94, 99) |
| **Confirmed Primary Source of Infection, N (%)** | | | | |
| Urinary tract | 52 (30) | 44 (39) | 5 (12) | 3 (18)  6 (35)  3 (18)  2 (12)  3 (18)  2 (11)  0 (0)  0 (0) |
| Intra-abdominal | 40 (23) | 22 (19) | 12 (30) |  |
| Pulmonary | 47 (27) | 23 (20) | 21 (51) |  |
| Soft-tissue/bone/joint | 28 (16) | 22 (19) | 4 (10) |  |
| Blood w/o another source | 10 (6) | 5 (4) | 2 (5) |  |
| Surgical site infection | 19 (11) | 15 (13) | 2 (11) |  |
| Surgical thoracic | 5 (3) | 1 (1) | 4 (10) |  |
| Other | 4 (3) | 3 (3) | 1 (2) |  |
| **Microbiology, N (%)** | | | | |
| Positive blood culture | 66 (40) | 44 (39) | 12 (29) | 10 (59)  6 (35)  5 (29)  12 (71) |
| Gram positive blood culture | 29 (18) | 17 (15) | 6 (15) |  |
| Gram negative blood culture | 36 (22) | 27 (24) | 4 (10) |  |
| Any positive culture | 113 (66) | 76 (67) | 25 (61) |  |

Note: data is count (percentage), unless otherwise specified by *median (1^st^ quartile, 3^rd^ quartile) unless otherwise stated; SBP, systolic blood pressure; HR, heart rate; RR, Respiratory Rate.
