# Supplementary material for: A hypolipoprotein sepsis phenotype indicates reduced lipoprotein antioxidant capacity, increased endothelial dysfunction and organ failure, and worse clinical outcomes
Source: Crit Care. 2021 Sep 17;25:341. doi: 10.1186/s13054-021-03757-5 (PMC8447561; doi:10.1186/s13054-021-03757-5)
Supplement: Supplementary file 9 — Additional file 9: Supplemental Table 2. Clinical management and outcomes for the derivation cohort. [file 13054_2021_3757_MOESM9_ESM.docx]

**Supplemental Table 2. Clinical management and outcomes for derivation cohort.**

| **Variable** | **All patients**  **(N = 172)** | **Rapid Recovery**  **(N=114)** | **CCI**  **(N=41)** | **Early Death**  **(N=17)** |
| --- | --- | --- | --- | --- |
| **Resuscitation Interventions** | | | | |
| Fluids in first 6 hours (mL), median (IQR) | 2000 (814, 3113) | 2000 (827, 3088) | 1272 (552, 3000) | 3000 (1000, 4000) |
| Fluids in first 24 hours (mL),  median (IQR) | 4000 (2500, 5500) | 3750 (2500, 5063) | 4135 (2175, 5580) | 5335 (3800, 8235) |
| Time to antibiotics (min), median (IQR) | 113 (75, 163) | 123 (81, 173) | 91 (63, 113) | 109 (65, 183) |
| Mechanical ventilation (mean, SD) | 84 (49) | 34 (30) | 36 (88) | 14 (82) |
| Vasopressors given (mean, SD) | 63 (37) | 25 (22) | 22 (54) | 16 (94) |
| Vasopressor duration (hrs), median (IQR) | 39 (21, 61) | 0 (0, 0) | 6 (0, 30) | 57 (46, 73) |
| Intubated at enrollment  (mean, SD) | 55 (32) | 21 (18) | 24 (59) | 10 (59) |
| **Admission Disposition, N (%)** | | | | |
| Ward | 54 (31) | 41 (39) | 11 (27) | 2 (12) |
| ICU | 118 (69) | 73 (61) | 30 (73) | 15 (88) |
| **Length of Stay, days (IQR)** | | | | |
| Hospital LOS | 11 (7, 20) | 9 (6, 16) | 29 (17, 38) | 7 (2,9) |
| ICU LOS | 4 (2,12) | 3(1, 5) | 19 (15, 25) | 6 (2,8) |
| **Clinical Disposition, N (%)** | | | | |
| *Good Disposition* | | | | |
| Home | 78 (45) | 71 (62) | 7 (17) | 0 |
| Rehab facility | 16 (9) | 10 (9) | 6 (15) | 0 |
| *Poor Disposition* | | | | |
| Nursing Home | 33 (19) | 30 (26) | 2 (5) | 1 (6) |
| Long-term acute care | 15 (9) | 3 (2) | 12 (30) | 0 |
| Hospice | 8 (5) | 0 | 7 (17) | 1(6) |
| In-hospital death | 0 | 0 | 7 (17) | 15 (88) |
| **Outcomes, N (%)** | | | | |
| 28 Day Mortality | 29 (17) | 1 (1) | 11 (27) | 17 (100) |
| 90 Day Mortality | 32 (19) | 3 (3) | 12 (29) | 17 (100) |
| 1-year Mortality | 43 (25) | 10 (9) | 16 (39) | 17 (100) |

Note: data is count (percentage), unless otherwise stated; **n=63; SBP, systolic blood pressure; HR, heart rate; RR, Respiratory Rate; SOFA, sequential organ failure assessment score; LOS, length of stay; ED, emergency department; ICU, intensive care unit
