# Supplementary material for: A hypolipoprotein sepsis phenotype indicates reduced lipoprotein antioxidant capacity, increased endothelial dysfunction and organ failure, and worse clinical outcomes
Source: Crit Care. 2021 Sep 17;25:341. doi: 10.1186/s13054-021-03757-5 (PMC8447561; doi:10.1186/s13054-021-03757-5)
Supplement: Supplementary file 10 — Additional file 10: Supplemental Table 3. Top features contributing to cluster discrimination between Hypolipoprotein and Normolipoprotein Cluster. [file 13054_2021_3757_MOESM10_ESM.docx]

**Supplemental Table 3.** **Unsupervised Clustering Significant Features.** Rates of the primary outcome by cluster (top) and significant unsupervised clustering elements for distinguishing Hypolipoprotein from Normolipoprotein cluster (bottom) in the derivation cohort.

| **Machine Learning Signature – Significant features contributing to cluster differentiation (Hypolipoprotein vs. Normolipoprotein).** | | |
| --- | --- | --- |
| **Feature Variable** | **Statistic** | **P-Value** |
| **HDL-C** | 7.532 | **<.0001** |
| **ApoA-I** | 7.285 | **<.0001** |
| **Total SOFA score** | -6.776 | **<.0001** |
| **Total cholesterol** | 6.700 | **<.0001** |
| **ICAM-1** | -5.855 | **<.0001** |
| **LDL-C** | 4.667 | **<.0001** |
| **Cardio SOFA** | -4.126 | **<.0001** |
| **Hepatic SOFA** | -3.228 | **0.0015** |
| **PON-1** | 3.162 | **0.0019** |
| **CNS SOFA** | -3.153 | **0.0019** |
| **Temperature** | 3.037 | **0.0028** |
| **Respiratory SOFA** | -2.904 | **0.0042** |
| **Renal SOFA** | -2.854 | **0.0049** |
| **Systolic Blood Pressure** | 2.687 | **0.0080** |
| **Coagulation SOFA** | -2.673 | **0.0083** |
