# Supplementary material for: A hypolipoprotein sepsis phenotype indicates reduced lipoprotein antioxidant capacity, increased endothelial dysfunction and organ failure, and worse clinical outcomes
Source: Crit Care. 2021 Sep 17;25:341. doi: 10.1186/s13054-021-03757-5 (PMC8447561; doi:10.1186/s13054-021-03757-5)
Supplement: Supplementary file 11 — Additional file 11: Supplemental Table 4. Demographics and disease severity for the replication cohort. [file 13054_2021_3757_MOESM11_ESM.docx]

**Supplemental Table 4. Demographics and disease severity for replication cohort.**

| **Variable** | **All patients**  **(N = 86)** | **Rapid Recovery**  **(N=45)** | | **CCI**  **(N=25)** | **Early Death**  **(N=16)** | **P-value^** |
| --- | --- | --- | --- | --- | --- | --- |
| **Demographics** | | | | | | |
| Age, in years* | 66 (57, 75) | | 65 (56, 74) | 65 (57, 75) | 69 (61, 74) | 0.592^a^ |
| Gender, male | 43 (50) | | 19 (42) | 17 (68) | 7 (44) | 0.113^b^ |
| Race, White | 45 (52) | | 24 (53) | 13 (52) | 8 (50) | 0.631^b^ |
| Black | 41 (48) | | 21 (47) | 12 (48) | 8 (50) |  |
| **Medications** | | | | | | |
| Statin use | 34 (40) | | 17 (38) | 11 (44) | 6 (40) | 0.914^b^ |
| **Initial Biomarkers and Organ Failure** | | | | | | |
| 1^st^ serum lactate (mmol/dL)* | 3.5 (2.6, 5.1) | | 3.2 (2.4, 4.8) | 3.4 (2.7, 4.4) | 4.2 (3.0, 6.8) | 0.200^a^ |
| 2^nd^ serum lactate (mmol/dL)*  - n =73 | 3.2 (2.2, 4.3) | | 2.75 (1.85, 4.00) | 2.5 (1.8, 4.1) | 4.2 (2.7, 6.9) | 0.028^a^ |
| Enrollment SOFA score | 7 (5, 10) | | 6 (4, 9) | 7 (5, 10) | 11 (8, 14) | **<0.001^a^** |
| Apache II Score | 18 (13, 26) | | 16 (11, 18) | 20 (15, 28) | 27 (24, 32) | **<0.001^a^** |

Note: data is count (percentage), unless otherwise specified by *median (1^st^ quartile, 3^rd^ quartile); COPD, chronic obstructive pulmonary disease; ESRD, end stage renal disease; HIV, human immunodeficiency virus; ^a^Wilcoxon rank-sum test; ^b^Pearson Chi-square test. ^ For the replication cohort, after Bonferroni adjustment for multiple tests, significant if p-values < 0.003 (0.05/15 tests) were considered significant.
